# Evaluation of ^99m^Tc-HYNIC-VCAM-1_scFv_ as a Potential Qualitative and Semiquantitative Probe Targeting Various Tumors

**DOI:** 10.1155/2018/7832805

**Published:** 2018-05-03

**Authors:** Xiao Zhang, Fan Hu, Chunbao Liu, Lianglan Yin, Yingying Zhang, Yongxue Zhang, Xiaoli Lan

**Affiliations:** ^1^Department of Nuclear Medicine, Union Hospital, Tongji Medical College, Huazhong University of Science and Technology, Wuhan 430022, China; ^2^Hubei Key Laboratory of Molecular Imaging, Union Hospital, Tongji Medical College, Huazhong University of Science and Technology, Wuhan 430022, China

## Abstract

Vascular cell adhesion molecule 1 (VCAM-1) is overexpressed in varieties of cancers. This study aimed to evaluate the application of a single chain variable fragment (scFv) of anti-VCAM-1 antibody labeled with ^99m^Tc as a possible imaging agent in several tumors. VCAM-1 scFv was labeled with ^99m^Tc using succinimidyl 6-hydrazinium nicotinate hydrochloride, and ^99m^Tc-HYNIC-VCAM-1_scFv_ was successfully synthesized with a high radiolabeling yield. VCAM-1 expression was evaluated in six cell lines by immunofluorescence staining. In vitro binding assays showed different binding affinities of ^99m^Tc-HYNIC-VCAM-1_scFv_ in different tumor cell lines, with high uptake in B16F10 melanoma and HT1080 fibrosarcoma cells, which was consistent with immunofluorescence staining results. In vivo SPECT planar imaging demonstrated that B16F10 and HT1080 tumors could be clearly visualized. Less intense uptake was observed in human SKOV3.ip ovarian tumor, and weak uptake was observed in human A375m melanoma, MDA-MB-231 breast cancer, and 786-O renal tumors. These findings were confirmed by biodistribution and immunofluorescence studies. High uptake by B16F10 tumors was inhibited by excess unlabeled VCAM-1_scFv_. ^99m^Tc-HYNIC-VCAM-1_scFv_, which selectively binds to VCAM-1, can provide a qualitative and semiquantitative method for noninvasive evaluation of VCAM-1 expression by tumors.

## 1. Introduction

Metastasis, one of the hallmarks of malignancy, remains a significant clinical obstacle to a favorable prognosis. Early, accurate diagnosis and targeted therapy are crucial. A prognostic tumor biomarker is very helpful for the diagnosis and targeted therapy of cancers [1]. Vascular cell adhesion molecule 1 (VCAM-1) is an immunoglobulin- (Ig-) like adhesion molecule with seven extracellular Ig domains. VCAM-1 is believed to be responsible for tumor proliferation and metastasis, and its levels correlate with prognosis [2]. It is expressed by multiple types of aggressive neoplasms, including those involving lung, prostate, breast, ovaries, and colon [3]. VCAM-1 has emerged as a target for therapy of these tumors [4]. Considering the aberrant expression of VCAM-1 in tumor biology, the development of noninvasive molecular imaging for VCAM-1 is crucial for better tumor diagnosis, prognosis, and therapy planning.

Multiple new techniques for targeting VCAM-1 have been developed in the past decades, including monoclonal antibodies, nanobodies, peptides, and single chain variable fragments (scFvs) [5–8]. As we know, intact monoclonal antibodies have strong binding affinity, but their large molecular weight leads to their slow clearance from blood and poor tissue penetration into tumors. [9]. In contrast, small peptides have the advantages of prompt excretion of unbound tracer and allow prompter imaging, but at the cost of low binding affinity [10]. Given all these considerations, small antibody fragments of moderate size and sufficient targeting ability are becoming attractive candidates for clinical application [11].

We previously prepared the scFv of anti-VCAM-1 (VCAM-1_scFv_) using the phage display method, which is a widely used process to obtain scFvs with high specificity [12, 13]. Due to the small molecular size (~28 kDa), scFv has the advantage of rapid clearance through renal excretion, lower concentration in liver, and stronger penetration into tumor tissues [14].

The aim of this study was to explore the possibility of a noninvasive and semiquantitative method for targeting VCAM-1 in tumors, which may allow early cancer diagnosis, more precise prognosis, and targeted treatment options. In this study, we radiolabeled VCAM-1_scFv_ with ^99m^Tc using succinimidyl 6-hydrazinium nicotinate hydrochloride (SHNH) to detect levels of VCAM-1 in several tumor models* in vivo*.

## 2. Materials and Methods

### 2.1. ^99m^Tc-6-Hydrazinonicotinamide- (HYNIC-) VCAM-1_scFv_ Labeling Procedure

SHNH (20 *μ*g, 69.8 nmol, Solulink, Inc., San Diego CA, USA) was added to the scFv (78.4 *μ*g, 28 nmol, Shanghai Raygene Biotech Company) and reacted in darkness overnight at 4°C. Afterwards, 100 *μ*L tricine (100 mg/mL, pH 5.2, Sigma/Aldrich, St. Louis MO, USA), 4 *μ*L SnCl_2_·2H_2_O (7 mg/mL, Sigma-Aldrich), and 500 *μ*L ^99m^TcO_4_^−^ solution (555 MBq, Beijing Atom High Tech, Beijing, China) were added to the reactions and incubated for 30 min at room temperature to prepare ^99m^Tc-HYNIC-VCAM-1_scFv_. The product was purified using a PD-10 gel column (General Electric, Fairfield CT, USA). The radiolabeled compound was analyzed by instant thin layer chromatography (ITLC) under identical conditions to calculate its radiolabeling efficiency, radiochemical purity, and* in vitro* stability (1, 3, 6, and 12 h in fetal bovine serum [FBS] and phosphate-buffered saline [PBS], *n* = 5 per group). Fifty percent acetonitrile and 0.01 M PBS were used as the developing solvent system.

### 2.2. Cell Culture

B16F10 and A375m melanoma cells, SKOV3.ip human ovarian cancer cells, and MDA-MB-231 human breast cancer cells were cultured in Dulbecco's modified Eagle's medium (DMEM) (Gibco, Carlsbad CA, USA). 786-O human renal cancer cells and HT-1080 human fibrosarcoma cells were maintained in Roswell Park Memorial Institute (PRMI-1640) and Minimal Essential Medium (MEM), respectively. All of the media were supplemented with 10% FBS (Gibco, Grand Island, NY, USA), 100 U/mL penicillin, and 100 *μ*g/mL streptomycin (Beyotime, Shanghai, China).

### 2.3. VCAM-1 Expression Confirmed by Immunofluorescence Staining

B16F10, HT-1080, SKOV3.ip, A375m, MDA-MB-231, and 786-O cells were digested, resuspended, and seeded in six-well plates at 1 × 10^6^ cells/well and incubated overnight. Then the samples were fixed with 4% paraformaldehyde at room temperature for 20 min and blocked in 1% bovine serum albumin (BSA) for 1 h, followed by incubation with VCAM-1 antibody (rabbit anti-VCAM-1, diluted 1 : 200, Abcam, Cambridge, MA, USA) at 4°C overnight. The next day, after incubation with fluorescent antibody (fluorescein isothiocyanate- [FITC-] labeled goat anti-rabbit IgG, diluted 1 : 50, Aspen, Wuhan, China) at 4°C for 30 min, the cells were stained with 4-6-diamidino-2-phenylindole (DAPI) for 5 min. Finally, the samples were observed under a confocal microscope (LSM 710; Zeiss, Oberkochen, Germany).

### 2.4. Cell Binding Assay

The binding affinity of ^99m^Tc-HYNIC-VCAM-1_scFv_ to B16F10, HT-1080, SKOV3.ip, A375m, MDA-MB-231, and 786-O cells was measured by a cell uptake assay. Briefly, the experiment was carried out in 24-well plates (2 × 10^5^ cells/well) and then incubated with 0.5 mL serum-free DMEM, PRMI-1640, or MEM containing ^99m^Tc-HYNIC-VCAM-1_scFv_ (2 nM) at 37°C for 1, 2, or 4 h, respectively. Thereafter, the cells were rinsed twice with 1 mL PBS and lysed with 1 N NaOH. The radioactivity in the cell lysate was counted using an automatic well-type gamma counter (PerkinElmer WIZARD2 2470, Shelton, CT, USA). For a blocking study, B16F10 cells were incubated with ^99m^Tc-HYNIC-VCAM-1_scFv_ (2 nM) at 37°C for 4 h in the presence of no other VCAM-1_scFv_, 100 nM unlabeled VCAM-1_scFv_, or 100 nM unlabeled HYNIC-VCAM-1_scFv_, and the radioactivity of the cell suspensions was measured.

### 2.5. Preparation of Tumor Models

All animal studies were performed in accordance with a protocol approved by the Institutional Animal Care and Use Committee (IACUC) of Tongji Medical College, Huazhong University of Science and Technology. Female BALB/c nude mice (3-4 weeks, Beijing HFK Bioscience Company, Beijing, China) were injected subcutaneously in the left shoulder with 5 × 10^6^ B16F10, HT-1080, A375m, MDA-MB-231, SKOV3.ip, or 786-O cells (*n* = 5 per group), suspended in 150 *μ*L PBS. The mice were used as models for* in vivo* SPECT planar imaging and biodistribution studies when the xenograft masses reached a size of 5 to 10 mm.

### 2.6. SPECT Planar Imaging

Imaging studies were performed in the tumor-bearing mice using SPECT (Symbia T6, Siemens, Erlangen, Germany) with a 3.0 mm pinhole collimator. Briefly, under isoflurane anesthesia, after intravenous injection of ^99m^Tc-HYNIC-VCAM-1_scFv_ (7.4–11.1 MBq), images were acquired at 1, 2, and 4 h postinjection. For the blocking study, B16F10 tumor-bearing mice were given a 50-fold excess dose of unlabeled VCAM-1_scFv_ 1 h prior to the injection of ^99m^Tc-HYNIC-VCAM-1_scFv_. The acquisition time was 10 min for each mouse.

### 2.7. Biodistribution Study

For biodistribution studies, five B16F10 tumor-bearing mice were injected with ^99m^Tc-HYNIC-VCAM-1_scFv_ (1.85 MBq) via tail vein and sacrificed at 1, 2, and 4 h postinjection. For the blocking study, B16F10 tumor-bearing mice (*n* = 5) were sacrificed at 1 h after the injection. The biological tissues of interest (i.e., blood, brain, myocardium, liver, spleen, lung, kidney, stomach, intestine, muscle, bone, and tumor) were removed, washed, and weighed, and their radioactivity was measured with decay correction using an automatic well-type gamma counter. The uptake in the tissues was expressed as a percentage of the injected dose per gram of tissue (% ID/g). Five groups of five mice, each group grafted with one of the five other tumors, were sacrificed 4 h postinjection of ^99m^Tc-HYNIC-VCAM-1_scFv_ (1.85 MBq), and the % ID/g was calculated as described above. Portions of the tumors, livers, and kidneys in the 4 h biodistribution groups were removed to check the expression of VCAM-1 with immunofluorescence staining. The results were analyzed using ImageJ software (version 1.46r, Wayne Rasband, National Institutes of Health, Bethesda, MD, USA).

### 2.8. Statistical Analysis

Statistical Package for the Social Sciences (SPSS) software (version 13.0, SPSS Inc., Chicago, IL, USA) and GraphPad Prism (version 5.0, San Diego, CA, USA) were applied in statistical analysis. All data are presented as the mean ± standard deviation (SD). Means were compared using Student's *t*-test with *P* < 0.05 being statistically significant.

## 3. Results

### 3.1. Radiolabeling Yield, Radiochemistry, and Stability

**Figure 1:**
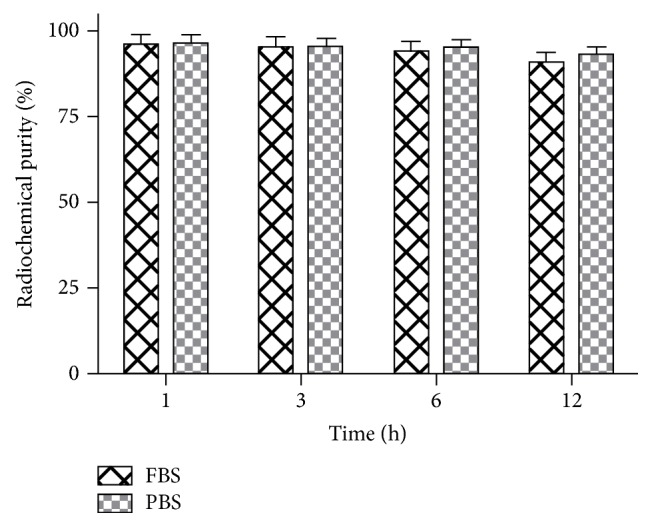
Stability of ^99m^Tc-HYNIC-VCAM-1_scFv_ in FBS and PBS at different time points. All data are expressed as the means ± SD (*n* = 5). FBS = fetal bovine serum. PBS = phosphate-buffered saline.

^99m^Tc-HYNIC-VCAM-1_scFv_ had a high radiolabeling yield of 81.5 ± 3.6% and a specific activity of 16.2 ± 1.1 MBq/nmol (*n* = 5). After purification, the radiochemical purity of ^99m^Tc-HYNIC-VCAM-1_scFv_ reached 96.5 ± 1.7% and was >90% at 1, 3, 6, and 12 h in FBS and PBS (Figure 1), indicating good stability* in vitro*.

### 3.2. VCAM-1 Expression In Vitro

**Figure 2:**
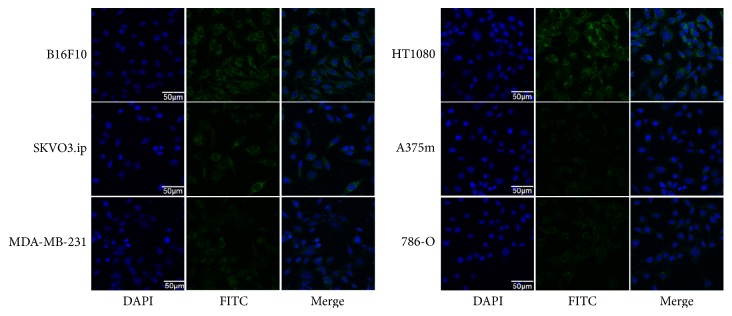
Immunofluorescence staining of six cancer cell lines. The cells were incubated with VCAM-1 antibody (green) and nuclei were stained with 4′,6-diamidino-2-phenylindole (DAPI). Representative images are displayed at the same scale (×600) (*n* = 5). DAPI = 4′,6-diamidino-2-phenylindole. FITC = fluorescein isothiocyanate.

The relative VCAM-1 expression levels in the cancer cell lines evaluated by immunofluorescence are shown in Figure 2. The results demonstrated strong intensities in B16F10 and HT1080 cells, a moderate intensity in SKOV3.ip cells, and low intensities in A375m, MDA-MB-231, and 786-O cells, which correlated with the VCAM-1 expression levels of these cells.

### 3.3. Cell Binding Assay

**Figure 3:**
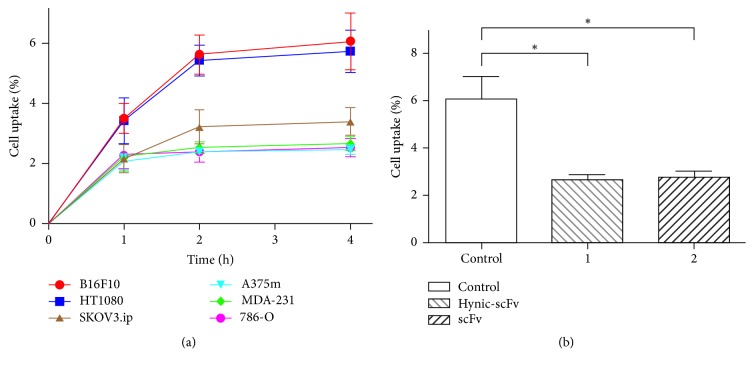
Cell uptake and blocking studies of ^99m^Tc-HYNIC-VCAM-1_scFv_* in vitro*. The ^99m^Tc-HYNIC-VCAM-1_scFv_ (2 nM) cell uptake studies (a) were performed on a series of cancer cell lines at serial time points. The comparisons of ^99m^Tc-HYNIC-VCAM-1_scFv_ uptake in the blocking experiments (b) with no other VCAM-1_scFv_, excess unlabeled VCAM-1_scFv_, and HYNIC-VCAM-1_scFv_. ^*∗*^*P* < 0.01. All data are expressed as mean ± SD in triplicate.

As shown in Figure 3(a), the uptake of ^99m^Tc-HYNIC-VCAM-1_scFv_ by B16F10 and HT1080 cells (2 × 10^5^) increased with time and reached a plateau (6.07 ± 0.55%, 5.73 ± 0.41%) at 4 h. The accumulation of radioactivity in SKOV3.ip cells (3.40 ± 0.26%, 4 h) had a moderate increase and the binding of ^99m^Tc-HYNIC-VCAM-1_scFv_ to A375m, MDA-MB-231, and 786-O cells remained relatively stable over time (2.47 ± 0.09%, 2.67 ± 0.13%, and 2.53 ± 0.18%, 4 h). These data reveal that ^99m^Tc-HYNIC-VCAM-1_scFv_ binds strongly to B16F10 and HT1080 cells, moderately to SKOV3.ip cells, and weakly to A375m, MDA-MB-231, and 786-O cells. The blocking study (Figure 3(b)) showed that the uptake of ^99m^Tc-HYNIC-VCAM-1_scFv_ in the presence of 100 nM unlabeled HYNIC-VCAM-1_scFv_ or 100 nM unlabeled VCAM-1_scFv_ in B16F10 cells was much lower than their corresponding nonblocked groups at 4 h (6.07 ± 0.55%, 2.67 ± 0.12%, and 2.77 ± 0.15%, *P* < 0.01). The blocking study demonstrates the specificity of ^99m^Tc-HYNIC-VCAM-1_scFv_ for VCAM-positive cells.

### 3.4. SPECT Planar Imaging

**Figure 4:**
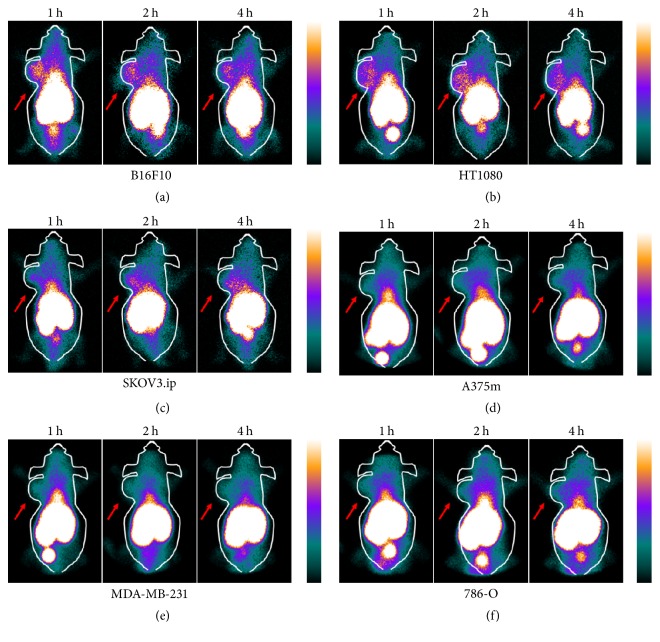
Representative SPECT planar imaging of ^99m^Tc-HYNIC-VCAM-1_scFv_ in six tumor models (*n* = 5 per group) obtained at 1, 2, and 4 h, respectively. Arrows refer to tumors.

**Figure 5:**
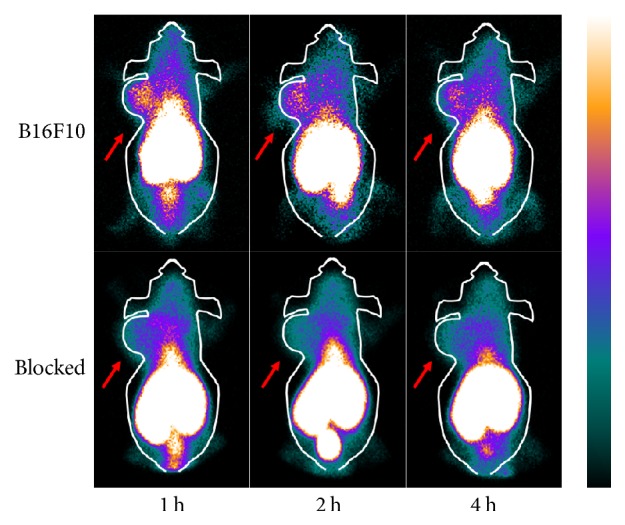
SPECT planar imaging of B16F10 tumor-bearing mice at the indicated time points after ^99m^Tc-HYNIC-VCAM-1_scFv_ injection, with/without preinjection of excess unlabeled VCAM-1_scFv_. Arrows indicate tumors.

B16F10 and HT1080 tumors images were clearly visualized, with high tumor-to-background contrast at all scan time points (3.20 ± 0.63, 3.90 ± 0.85, and 3.21 ± 1.05 for B16F10; 3.39 ± 0.65, 3.28 ± 0.84, and 3.13 ± 0.63 for HT1080, Figures 4(a) and 4(b)). We chose contralateral limb of the tumor as background value. Weaker uptake was observed by SKOV3.ip tumor (2.75 ± 0.57, 1 h, Figure 4(c)), and the probe uptakes in A375m, MDA-MB-231, and 786-O tumors (Figures 4(d), 4(e), and 4(f)) were indistinguishable from background (1.85 ± 0.32, 1.84 ± 0.75, and 1.77 ± 0.47, 1 h). As shown in Figure 5, the accumulation of radioactivity in B16F10 tumors clearly decreased in the presence of excess unlabeled VCAM-1. These results indicate that ^99m^Tc-HYNIC-VCAM-1_scFv_ can specifically target VCAM-1-positive tumors. In addition, the images of the kidneys and the livers were visualized clearly, which confirmed that in vivo clearance of the probe is mainly through the renal and hepatic routes.

### 3.5. Biodistribution Study

**Figure 6:**
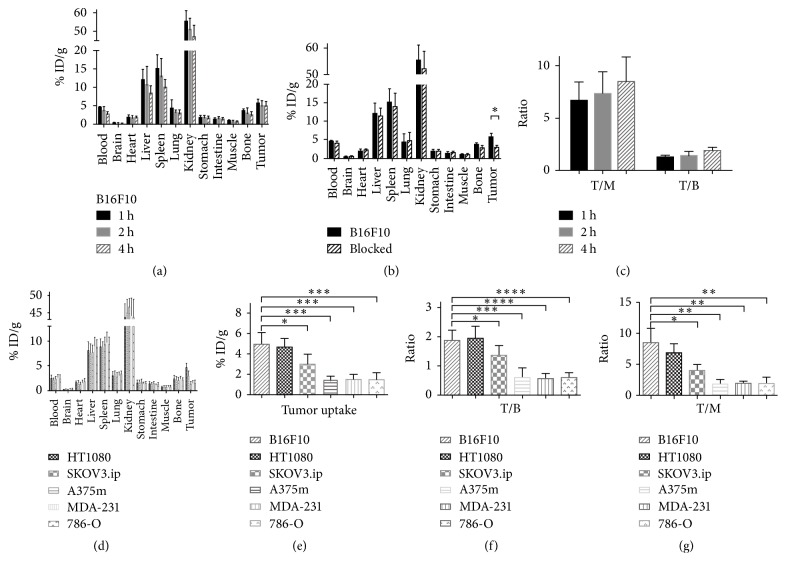
Tissue biodistribution of ^99m^Tc-HYNIC-VCAM-1_scFv_ in tumor xenografts. Biodistribution of ^99m^Tc-HYNIC-VCAM-1_scFv_ was assessed in mice bearing B16F10 tumors at 1, 2, and 4 h postinjection ((a), *n* = 5). The blocking study with excess VCAM-1_scFv_ was performed at 1 h after ^99m^Tc-HYNIC-VCAM-1_scFv_ injection in B16F10 tumor-bearing mice (b). Tumor-to-blood (T/B) and tumor-to-muscle (T/M) ratios in mice bearing B16F10 tumors at the indicated time points (c). Biodistribution was similarly examined in five tumor models at 4 h postinjection (d). The comparison of tumor uptake (e), T/B (f), and T/M (g) in the different tumors. ^*∗*^*P* < 0.05, ^*∗∗*^*P* < 0.01, ^*∗∗∗*^*P* < 0.001, and ^*∗∗∗∗*^*P* < 0.0001.

We also assessed tumor targeting and nontumor tissue distribution of ^99m^Tc-HYNIC-VCAM-1_scFv_ in the six tumor models (Figure 6). In B16F10 tumor models (Figure 6(a)), highest accumulation was noted in the kidneys at all time points, and the radioactivities in kidneys decreased steadily. The results indicated urinary system was the main pathway of ^99m^Tc-HYNIC-VCAM-1_scFv_ excretion. The B16F10 tumor uptakes were 5.51 ± 0.37% ID/g, 5.04 ± 0.61% ID/g, and 4.93 ± 0.52% ID/g at 1, 2, and 4 h postinjection, respectively, and tumor-to-blood (T/B) and tumor-to-muscle (T/M) ratios increased over time from 1.25 ± 0.08 and 6.68 ± 0.79 at 1 h postinjection to 1.88 ± 0.17 and 8.47 ± 1.05 at 4 h postinjection in B16F10 xenograft mice (Figure 6(c)), which bodes well for the application of ^99m^Tc-HYNIC-VCAM-1_scFv_ as an* in vivo* molecular imaging agent. As expected, the tumor concentration of ^99m^Tc-HYNIC-VCAM-1_scFv_ in the blocked mice was significantly lower than that in the unblocked mice (2.92 ± 0.26% ID/g versus 5.51 ± 0.37% ID/g, *P* < 0.001, Figure 6(b)) at 1 h, while the uptake in nontumor tissues was not significantly reduced by the blocking dose, suggesting that nontumor tissues did not express significant VCAM-1 and took up the tracer nonspecifically. The uptakes of ^99m^Tc-HYNIC-VCAM-1_scFv_ in HT-1080, SKOV3.ip, A375m, MDA-MB-231, and 786-O tumor models were 4.65 ± 0.39% ID/g, 2.99 ± 0.44% ID/g, 1.33 ± 0.22% ID/g, 1.49 ± 0.23% ID/g, and 1.47 ± 0.31% ID/g at 4 h postinjection (Figures 6(d) and 6(e)), respectively, which were in agreement with* in vivo *images. The ratios of T/B and T/M (Figures 6(f) and 6(g)) were also similar.

**Figure 7:**
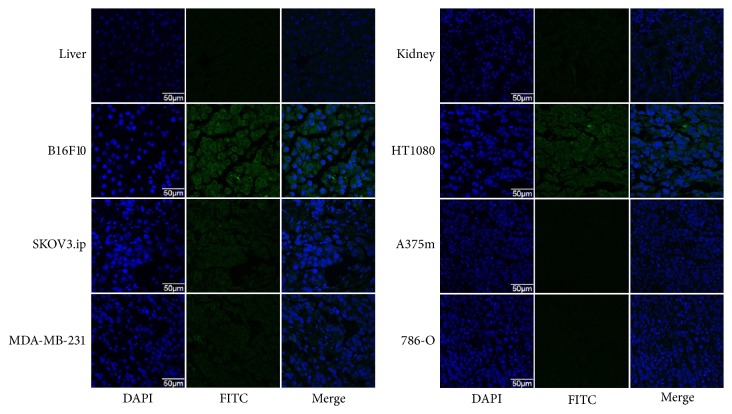
Immunofluorescence staining of kidneys, livers, and various tumors. The tissues were incubated with VCAM-1 antibody (green), followed by staining with DAPI. Representative images are displayed at the same scale (×600, *n* = 5).

**Figure 8:**
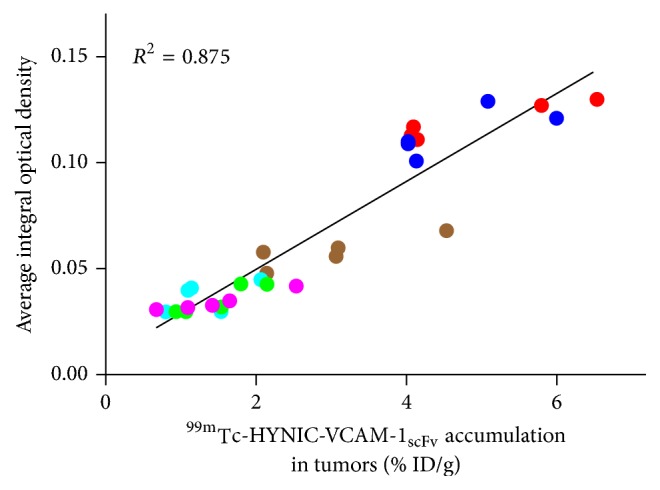
Relationship between the tumor uptake and mean integral optical density of VCAM-1 expression (*n* = 5 per group).

As shown in Figure 7, immunofluorescence staining of the kidneys and liver showed relatively low signals, indicating that these tissues did not express VCAM-1 significantly, again showing that high uptake in the kidneys and liver was unrelated to specific binding to VCAM-1 in these organs and largely attributed to the clearance of the probe. The immunofluorescence intensities of the tumor tissues (Figure 7), which were extracted from different tumor-bearing mice of the 4 h biodistribution groups, further validated the different VCAM-1 expression levels of six tumor models. ^99m^Tc-HYNIC-VCAM-1_scFv_ accumulation in the tumors significantly correlated well with average integral optical density of VCAM-1 expression (Figure 8, *R*^2^ = 0.875, *P* < 0.0001), which suggested the possibility of semiquantitative evaluation for VCAM-1 noninvasively* in vivo*.

## 4. Discussion

Recently, strategies for targeting VCAM-1 [5–8], such as ^18^F labeled nanobodies and ^111^In labeled peptides, have been investigated. VCAM-1 expressed in atherosclerosis has been the main target of research. For the availability and low costs, ^99m^Tc is an ideal radionuclide for radiopharmaceutical synthesis and has been used more widely than ^18^F and ^111^In in clinical applications. Therefore, we used ^99m^Tc-HYNIC-VCAM-1_scFv_ to target VCAM-1 in various tumors to assess the binding affinity and characteristics of VCAM-1_scFv_.

^99m^Tc has a half-life of 6 h, which is well matched with the relatively short physiological blood half-life of VCAM-1_scFv_, making the probe's clinical translation feasible in future [15]. In addition, HYNIC-NHS, as a bifunctional chelating agent, participates in the radiolabeling of ^99m^Tc with peptides or antibodies, which is easily prepared, with a high radiolabeling yield, radiochemical purity, and stability [16, 17]. As an alternative to PET, we sought to evaluate the feasibility of a ^99m^Tc-HYNIC-VCAM-1_scFv_ as a SPECT tracer to target VCAM-1, which is much cheaper and more easily available. In the uptake studies, six cell lines with different VCAM-1 expression levels confirmed by immunofluorescence staining showed corresponding binding affinities of the radiolabeled VCAM-1_scFv_. The uptake value in B16F10 cells (VCAM-1 positive) was effectively blocked by an excess of unlabeled VCAM-1_scFv_, further verifying the specificity of ^99m^Tc-HYNIC-VCAM-1_scFv_ to VCAM-1* in vitro*.

The uptake pattern and blocking studies in the different cell lines closely correlated with the SPECT planar imaging and biodistribution study of xenograft models. In the imaging of B16F10 and HT1080 xenograft mice, tumors were visualized clearly from normal tissues as early as 1 h after injection of ^99m^Tc-HYNIC-VCAM-1_scFv_. Based on the high tissue penetrability of small antibody fragments, the probe can reach the tumor site more quickly [18]. It is significantly different from tumor imaging with intact monoclonal antibodies, mainly due to slow clearance of the larger tracers from the blood pool [19]. Moderate uptake was seen in SKOV3.ip tumors, measuring 2.99 ± 0.44% ID/g at 4 h. In contrast with VCAM-1-^111^In peptide distribution in omentum of SKOV3ip1 cells (about 2% ID/g), ^99m^Tc-HYNIC-VCAM-1_scFv_ has a relatively higher binding affinity with SKOV3.ip tumors [8]. No obvious tracer uptake could be seen in the imaging of A375m, MDA-MB-231, and 786-O tumor models, which are consistent with the lack of VCAM-1 expression in these tumor tissues and also confirmed by immunofluorescence staining of the tissues. High T/M ratios in B16F10 and HT1080 xenografts (8.47 ± 1.05 and 6.89 ± 0.64) were observed at 4 h postinjection, showing ideal contrast for imaging of these tumors. These results suggest the targeting ability and specificity of the probe* in vivo*.

As the main excretory organs, the kidneys showed the highest accumulation in the biodistribution study, which is in agreement with Broisat et al. [20]. This is due to the small molecular weight (~28 kDa) of scFv, which is below the cut-off for renal clearance. Immunofluorescence staining showed liver and kidneys do not express significant VCAM-1, demonstrating that the accumulations in the kidneys and livers is mainly related to the excretion mode. The immunofluorescence intensity of VCAM-1 has a relationship with the radioactivity accumulated in tumors, which indicates the feasibility of achieving semiquantitative evaluation of VCAM-1 expression* in vivo*.

There are various methods to detect protein expression using molecular biological techniques [21], such as immunohistochemistry, immunofluorescence, western blotting, and ELISA. Among them, ELISA is mainly applied for the detection of secretory proteins [22]. Although the specificity of western blotting is high, the procedure is complex. This research focuses on the study of immunofluorescence and radioimmunoassay, which are both based on the antigen-antibody reaction. VCAM-1 expression detected by ^99m^Tc-HYNIC-VCAM-1_scFv_ relies on the binding of the radioactive antibody fragment (VCAM-1_scFv_) with the antigen. After the binding, the radioactivity uptake is measured to quantitate the antigen expression [23]. This technique is easy to perform with high sensitivity. Immunofluorescence, which makes use of labeling antibodies with fluorescent substances, has gained more and more attention due to its high sensitivity and superiority of obtaining anatomic and physiological information. However, it needs to get specimen which is invasive and hard to repeat in the living body, and it also has some shortcomings, such as inadequate penetration depth [24]. In contrast, PET and SPECT imaging with the ability to image the living human body deeply, are more advantageous in clinical applications. The results of immunofluorescence in our study are consistent with radioimmunoassay, indicating that ^99m^Tc-HYNIC-VCAM-1_scFv_ has the potential to be used to detect VCAM-1 noninvasively and repeatedly* in vitro* and* in vivo*.

There are several issues that need to be pointed out in this study. First, high activity in blood was also observed, which renders T/B ratios (1.25 ± 0.08) not ideal in VCAM-1 positive tumor models at 1 h. Fortunately, the accumulation in blood decreased rapidly (from 4.54 ± 0.13% ID/g at 1 h to 2.67 ± 0.32% ID/g at 4 h) and the T/B value increased steadily. Second, although lower accumulation was seen in liver than that with intact monoclonal antibodies [25], the liver retained high amounts of activity after ^99m^Tc-HYNIC-VCAM-1_scFv_ administration. This will interfere with imaging of lesions in the liver and surrounding tissues. Further studies will focus on modifying and optimizing the probe to minimize its blood and liver accumulation.

## 5. Conclusion

We successfully labeled an scFv-based probe, ^99m^Tc-HYNIC-VCAM-1_scFv_, that specifically binds to VCAM-1. We identified different expression levels of VCAM-1 with SPECT planar imaging of corresponding tumor lesions, which potentially provides a qualitative and semiquantitative method for noninvasive evaluation of VCAM-1 expression* in vivo*.
